# Update on clinical radiobiology

**DOI:** 10.2349/biij.2.1.e22

**Published:** 2006-01-01

**Authors:** N Chawapun

**Affiliations:** Division of Therapeutic Radiology and Oncology, Faculty of Medicine, ChiangMai University, Chiang Mai, Thailand

**Keywords:** Radiobiology, molecular targeted therapy, tumour microenvironment, molecular imaging

## Abstract

Radiation therapy is an important local cytotoxic modality for cancer treatment whose aim is to control the disease while minimising damage to normal tissue. The combination of different treatment modalities offers a more effective cure and reduction in normal tissue toxicity. However, the differences in genetic profiles can cause diverse treatment outcomes. Multidisciplinary research, where technologies and knowledge from different areas are integrated, is necessary to design the optimal regimen for individualised cancer treatment. This paper offers an overview of some new cancer treatment strategies; the impact of molecular imaging on radiation oncology; and a computer simulation model to optimise treatment planning based on patient information. It briefly discusses molecular targeted therapy, tumour microenvironment and bioreductive agents, and evidence for making individualised medicine a reality. Using DNA microarrays and proteomic technologies, information on defined molecular targets and genetic profiling for individual patients can be obtained and new algorithms for radiation oncology-related diagnosis, treatment response and prognosis can be developed.

## INTRODUCTION

The treatment of cancer currently uses different modalities, either alone or in combination, for more effective cures and to avoid unwanted effects. Experimental and theoretical studies in radiation biology and other related fields have substantially grown to improve the field of radiotherapy which has undergone a marked development as a consequence of new biological and technological knowledge and abilities.

Different cancers behave and respond to treatment differently. Therefore, we need to understand the molecules involved and their functions, such as intracellular and intercellular signalling cascades that regulate radiation sensitivity and resistance of both tumour and normal cells. As basic molecular biology techniques have been progressing rapidly and the human genome project has completed mapping and sequencing all the genes in humans, the information gained will greatly impact cancer treatment.

Individualised cancer treatment, which is the goal of the next revolution in radiation oncology, can be achieved with intense approaches into the field of DNA repair, cell cycle control and signalling transduction, tumour microenvironment and molecular targeting in radiation oncology.

## MOLECULAR TARGETING IN RADIATION ONCOLOGY

Targeted therapy is a medication or chemical that targets a specific pathway in the growth and development of cancer. There are several classes of targeted therapies [1] such as tyrosine kinase receptor inhibitors, angiogenesis inhibitors, proteosome inhibitors, and immunotherapy.

### Tyrosine kinase receptor inhibitors

**Figure 1:**
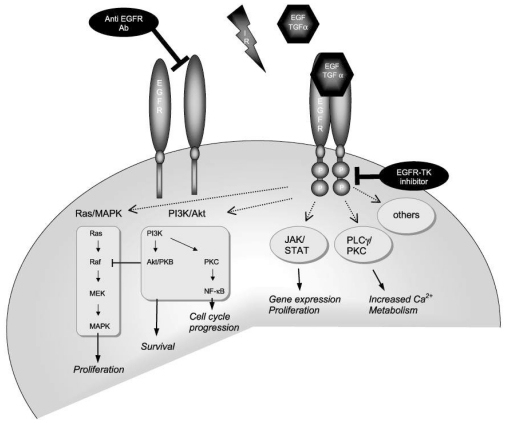
Binding of a ligand to the EGFR initiates a cascade of cellular reactions (from Baumann [2]).

Cell growth in normal tissue is controlled by balancing growth-promoting and growth-inhibiting factors. When the balance in malignant tumour cells is disturbed, the cells can proliferate without control. A key driver for growth is the epidermal growth factor (EGF) and its receptor [epidermal growth factor receptor (EGFR)]. EGFR is a member of a family of four receptors, i.e., EGFR (HER1/ErbB1), ErbB2 (HER2/neu), ErbB3 (HER3), and ErbB4 (HER4). These proteins are located in the cell membrane and each has a specific external ligand binding domain, a helical transmembrane domain, and an intracellular domain with tyrosine kinase enzyme activity [2,3]. As shown in Figure 1, the extracellular part contains two cysteine-rich domains that can bind a number of growth factors [2]. Activation of EGFR following the growth factor (ligand) binding leads to homodimerisation of the receptor with another EGFR, or heterodimerisation with another member of the EGFR family. Receptor dimerisation initiates a signalling cascade that results in protection from apoptosis and stimulation of cell proliferation, angiogenesis, cell differentiation and cell migration. EGFR intracellular signalling is mainly through two downstream pathways, the Ras-Raf-mitogen-activated protein kinases (MAPK) and the phosphatidylinositol 3-kinase (PI3K)/Akt pathways [2,3]. The quick-time animation of EGFR and its signalling cascade are available online [4].

The EGFR gene is a protooncogene and plays a key role in the development of human tumours. EGFR activation occurs due to EGFR gene amplification, gene mutation, or through growth factor overproduction. EGFR status can be determined by measuring protein expression, mRNA expression, gene amplification or gene mutation, but the most widely used assay is immunohistochemistry that measures protein expression [3].

The reasons for targeting EGFR in anticancer therapy are: the importance of EGFR signalling in the development of tumours; the association between EGFR overexpression and poor prognosis in many cancers; and the distinct mechanism of action of anti-EGFR approaches versus conventional methods. Currently, two approaches are in clinical development and clinical trials: the use of monoclonal antibodies (mAbs) directed against the external domain of the receptor, e.g., cetuximab, ABX-EGF, EMD72000; and the use of small molecule inhibitors of EGFR tyrosine kinase enzyme that competes with the ATP binding site of the intracellular domain of the EGFR and blocks intracellular signalling, e.g., ZD1839, OSI-774, CI-1033, EKB-569, GW572016 [5]. Trastuzumab or NO34 is the monoclonal antibody inhibitor for another family member, HER2/neu, that is used in breast cancer with overexpression of HER2 protein.

Other approaches in pre-clinical development for targeting EGFR are: anti-sense oligonucleotides, EGFR-directed vaccines, and immunoconjugates of antibodies coupled to a radioactive isotope or cytotoxin [3]. It is, therefore, classified as immunotherapy. Pre-clinical and clinical studies have shown that different inhibitors have in-vitro and in-vivo activity that potentiates the effects of cytotoxic agents and radiation. The addition of ZD1839 to cisplatin-5FU affects signalling pathways that control cell proliferation, apoptosis and DNA repair in human head and neck cancers' cell lines [6]. Caspase-3 activity was increased in the drug combination exposure, while ZD1839 alone induced G0/G1 arrest accompanied by an increase in G1 cell cycle regulator, p21 and p27, and Bax; and a decrease in Bcl-2, Akt phosphorylation and DNA-PK. The study on human breast cancer cell lines also showed a synergistic interaction between ZD1839 and chemotherapeutic agents in anti-proliferative action and the delayed repair of DNA damage [7]. The enhancement of tumour radioresponse through the modulation of DNA repair was reported [8,9].

The literature review on the role of EGFR-TK signalling in tumour response to radiation therapy indicates that EGFR-TK activity in tumours can block the cytotoxic effects of radiation therapy and enhance tumour repopulation, resulting in the failure of local tumour control [10]. Combining ZD1839 with radiation therapy can improve responses in non-small cell lung cancer, head and neck cancers, and other solid tumours. It has been hypothesised that exposure to ionising radiation activates the EGFR and subsequently activates the downstream signalling pathway involving the protective stress response. The treatment with the tyrosine kinase inhibitor can prevent radiation-induced EGFR autophosphorilation. Clinical studies reveal that it is the mutations within the EGFR-TK domain that predict the response to the EGFR inhibitor; not the level of tumour EGFR expression [11]. Further studies are required to identify patients who are likely to benefit from this therapy.

### Angiogenesis inhibitors

**Figure 2:**
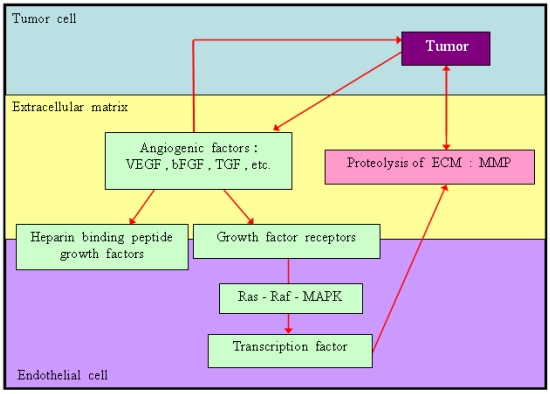
Illustration of the angiogenesis signalling cascade (adapted from Eckhardt [13]).

Angiogenesis is the formation of a new blood vessel from an existing vasculature. It occurs infrequently in normal adults, except during times of wound healing, inflammation, ovulation, pregnancy or ischemia. Tumour angiogenesis is the proliferation of a network of blood vessels that penetrates into cancerous growths, supplying nutrients and oxygen, and removing waste products. Angiogenesis is regulated by activator and inhibitor molecules, which activate certain genes in the host tissue [12]. Figure 2 illustrates the angiogenesis signalling cascade. The two most important angiogenesis-stimulating molecules, vascular endothelial growth factor (VEGF) and basic fibroblast growth factor (bFGF), are released by tumour cells into the surrounding tissue. The binding of the factor to its receptor at an endothelial cell surface activates the signalling pathway, leading to new endothelial cell growth. To form the new blood vessels, the activated endothelial cells produce the degradative enzymes, matrix metalloproteinases (MMPs), which are then released into the surrounding tissue to break down the extracellular matrix. This enables the endothelial cells to migrate, divide and then organise to form a network of blood vessels. Cells transformed by RAS, MYC, RAF, c-erbB-2, c-JUN or SRC have a strong angiogenic phenotype, either through the up-regulation of pro-angiogenic factors or reciprocal down-regulation of anti-angiogenic factors. It has been postulated that inhibiting angiogenesis can slow down or prevent the growth of cancer cells. The naturally occuring proteins that inhibit angiogenesis are angiostatin, endostatin and thrombospondin. Angiogenesis inhibitors can be categorised by their mechanism of action. The first category includes molecules that inhibit angiogenesis directly by inhibiting endothelial cell function, promoting apoptosis of endothelial cells, or interacting with integrin to promote the destruction of proliferating endothelial cells. The second category consists of molecules that block the angiogenesis signalling cascade and includes drugs that interfere with endothelial cell receptors or inhibit the production of pro-angiogenic factors. The third category includes molecules that block the breakdown of the extracellular matrix by inhibiting the activity of MMPs. The last category consists of inhibitors that involve, either a non-specific mechanism of actions or mechanisms that are not clearly understood. Table 1 shows some of the angiogenesis inhibitors that are being tested in cancer patients.

**Table 1:** The angiogenesis inhibitors that are being tested in cancer patients.

**Category**	**Mode of action**	**Drug(s)**
Inhibit angiogenesis directly	Inhibits the growth of endothelial cells	Endostatin
		EMD121974
		TNP-470
		Squalamine
	Causes apoptosis of endothelial cells	Combretastatin 4
	Promotes the destruction of proliferating endothelial cells by interacting with integrin	Medi-522
Block the angiogenesis signalling cascade	Inhibits the production of bFGF and VEGF	Interferon-alpha
	Blocks the binding to the receptor	Anti-VEGF antibodies
		(Bevacizumab or Avastin^TM^)
		SU5416
		SU6668
		PTK787/ZK22584
Block extracellular matrix breakdown	Inhibits the activity of MMPs	Marimistat
		AG3340
		COL-3
		Neovastat
		BMS-275291
Non-specific mechanism of action	Inhibits calcium uptake	CAI
	Up-regulates interferon-gamma and IP-10	Interleukin-12
	Unknown	IM862

Information on the status of anti-angiogenic clinical trials can be accessed at the National Cancer Institute website (http://www.cancer.gov/clinicaltrials/digestpage/angiogenesis-inhibitors). AEE788, a potent combined inhibitor of both EGF and VEGF receptor and a tyrosine kinase family member, is in Phase I clinical trials as it inhibits EGFR/ErbB2 phosphorylation-mediated proliferation in in-vitro studies and demonstrates anti-angiogenic effects in a VEGF-driven animal model [14].

### Proteosome inhibitors

Proteosome or a protein shredder is an enzyme complex in the cell, which is responsible for breaking down proteins. Many different proteins are destroyed by the proteosome when they are no longer needed, including signalling proteins, enzymes and structural proteins [15]. Disruption of this system can transform the cell. The important proteins that are substrates of proteosome include the inhibitor of nuclear factor kappa B (NF-κB;IκB), p53 tumour suppressor, the cyclin-dependent kinase inhibitors p21 and p27, and the proapoptotic protein Bax [16]. The molecular targets of proteosome inhibitors and their contribution to anti-tumour effect are summarised in Table 2. Proteosome inhibitors induce apoptosis, reverse drug resistance and affect the cell microenvironment by blocking cytokine circuits, cell adhesion and angiogenesis. Phase III clinical trials have been conducted for bortezomib, a new class of proteosome inhibitors, and the impressive result, as compared with high-dose dexamethasone in multiple myeloma treatment, was reported by the Assessment of Proteosome Inhibition for Extending Remission (APEX) group [17]. However, adverse events, i.e., peripheral neuropathy, cerebrovascular accident and grade 3 abdominal pain, were reported in patients with metastatic colorectal cancer during Phase II trials of this drug [18]. Phase I and II trials of bortezomib, in combination with other agents, have been conducted.

**Table 2:** Molecular targets of proteosome inhibitors [16]

**Target**	**Sequelae of proteosome inhibition**	**Contribution to anti-tumour effect**
NFκB*	Stabilisation of IκB, which inhibits nuclear translocation of NFκB	Decreases NFκB-dependent transcription of genes important in tumour cell survival, proliferation, invasion and metastasis, and angiogenesis
p53	Accumulation of p53 protein by inhibition of proteosome-mediated p53 degradation	Increases p53-dependent transcription of cell cycle inhibitors (p21) and proapoptotic factors (Bax)
p21 and p27	Accumulation of both p21 and p27, and increased transcription of p21 through accumulation of p53	Induces G1/S cell cycle arrest and apoptosis
Bax	Accumulation of Bax by inhibition of proteosome mediated Bax degradation and through increased p53-mediated transcription	Increases Bax interaction with Bcl-2 and Bcl-xL, promoting release of mitochondrial cytochrome cand apoptosis
p44/42MAPK	Transcriptional activation of the MKP-1 phosphatase, leading to p44/42 dephosphorylation andinactivation	Down-regulates p44/42-dependent cell proliferation and survival signals, and possibly angiogenesis
tBid	Accumulation through decreased proteosomal degradation	tBid induces conformational changes in Bak, promoting mitochondrial release of cytochrome c
Smac/Diablo	Accumulation through decreased proteosomal degradation	Smac and Diablo bind to and inhibit members of the XIAP protein family

* NFκB, nuclear factor κB; IκB, inhibitor of nuclear factor κB; MAPK, mitogen activated protein kinase; Diablo, direct IAP binding protein with low pI; Smac, Second mitochondria-derived activator of caspase; XIAP, X-chromosome-linked inhibitor of apoptosis.

### Immunotherapy

In targeted immunotherapy, the inhibitors bind to their targets. This does not interfere with the growth signal, but triggers the immune signal leading to a series of anti-tumour immune reactions in the body, causing the destruction of the tumour cells. The dual attack on tumour cells can be achieved by using radioimmunotherapy agents. The immunotherapy drugs are chemically attached to the radioactive substances, taking advantage of both the anti-tumour immune response and anti-tumour radiation reaction. Recently developed approaches in this field are classified as strategies based on cytokines, dendritic cell, vaccination, and targeting toll-like receptor [19].

## TUMOUR MICROENVIRONMENT

Tumour microenvironment is the characterisation of the physiological and metabolic conditions within solid tumors, whether primary or metastatic site. In most tumours, it shows low glucose concentration, high lactate concentration, low extracellular pH and low oxygen tensions [20]. The major factors causing tumour hypoxia (O_2_ tension ≤ 2.5 mm Hg) are abnormal structure and function of microvessels supplying tumours; large diffusion distances between blood vessels and tumour cells; and low oxygen transport capacity of blood [21]. Besides a reduction in the generation of free radicals, which is the direct mechanism, the alterations in gene expression as well as the genetic instability induced by hypoxia may increase resistance to therapy and add to the development of a more aggressive tumour phenotype [22]. Hypoxia-induced alterations in gene expression are controlled by a number of oxygen-regulated transcription factors, among which hypoxia-inducible factor 1 (HIF-1) and nuclear factor kappa B (NF-κB) dependent pathways are important. Two main strategies to overcome tumour hypoxia are to increase the delivery of oxygen or use oxygen-mimetic drug, and to exploit the environmental condition for targeted therapy [23]. The use of hyperbaric oxygen, electron-affinic radiosensitisers and nitroimidazole compound comprises the first strategy, while the use of bioreductive drugs and hypoxia-targeted suicide gene therapy leads to activated cytotoxic agents specifically at the tumour site. As patient compliance is a problem, only some nitroimidazole compounds such as nimorazole were reported to significantly improve the effect of radiotherapy in supraglottic, larynx and pharynx carcinomas [24]. The latter class of anti-tumour pro-drugs exploits the ability of solid cancers to carry out reductive metabolism, leading to the production of a cytotoxic species which can then damage and kill the malignant cells. [25] The rationale for clinical use is that the bioreductive metabolism reaction, especially when catalyzed by one-electron reductases, is oxygen sensitive – only occurring in the absence of oxygen. Therefore, bioreductive drug activation is favoured in many solid cancers containing hypoxic tumour cells because of insufficient and aberrant vasculature. Numerous compounds are currently at various stages of drug development, but mitomycin C and tirapazamine have been impressive in the clinic. Tirapazamine (1,2,4-benzotriazin-3-amine 1, 4-dioxide) becomes activated by NADPH: cytochrome 450 reductase to give cytotoxic metabolites. The suggested mechanism of action of tirapazamine is DNA strand break.

Radiosensitisation, through improvements in tumour oxygenation/hypoxic cell sensitisation, has limited success through the use of hyperbaric oxygen, electron-affinic radiosensitisers, and mitomycin. Phase I, II and III trials with tirapazamine in combination with cisplatin for the treatment of solid tumours, including non-small cell lung cancer, breast cancer, head and neck cancer, and melanoma, have been conducted. Tirapazamine as an adjuvant to radiotherapy for head and neck cancer, cervical cancer and glioblastoma multiforme is in Phase II trials.

## IMPACT OF MOLECULAR IMAGING ON RADIATION ONCOLOGY

Molecular imaging has two basic applications: diagnostic imaging, to determine the location and extent of targeted molecules; and therapy, to treat specific disease-target molecules by adding a therapeutic agent onto the probe.

In the last decade, substantial technological progress has led to major developments in radiation oncology. The new algorithms for three-dimensional reconstructions of anatomy and dose calculation enable the delivery of radiation treatment with high geometric precision, i.e., stereotactic radiotherapy (SRT), stereotactic radiosurgery (SRS), and intensity-modulated radiotherapy (IMRT). Therefore, more accurate tumour identification and delineation are required. The International Commission on Radiation Units and Measurement (ICRU) defines the concept of target volume for radiation treatment as: gross tumour volume (GTV), the volume that includes the demonstrable extent and location of the primary tumour, regional lymph nodes and distant metastases; clinical target volume (CTV), the volume that includes GTV and/or subclinical disease; and planning target volume (PTV) that includes any geometric uncertainties and set up margins [26]. The target volume definition is currently based on computed tomography (CT) and magnetic resonance imaging (MRI). The information obtained is only morphological. However, in recent years, new methods for tumour visualisation have begun to impact radiation oncology [27,28]. Techniques such as positron emission tomography (PET), single-photon emission computed tomography (SPECT) and magnetic resonance spectroscopy (MRS) permit the visualisation of biological pathways of tumours and offer additional information about metabolism, physiology and molecular biology of tumour tissue. This new class of images, showing specific biological events, complements the anatomic information from traditional radiological techniques. The most popular PET tracer is fluorine-18 fluorodeoxyglucose (FDG). FDG is a glucose analog that is transported into cells on the gluc-1 transporter. Gluc-1 is overexpressed in cancer and responsible for the high accumulation of F-18 FDG in metastatic cells. FDG is phosphorylated by hexokinase after entering the cell and trapped inside by the change in electrical charge. Another tracer is a thymidine derivative, F-18 fluorodeoxythymidine (FLT), that is phosphorylated by TK-1, an enzyme that is highly expressed in rapidly proliferating malignant cells during DNA synthesis.

Clinical studies on the integration of PET in target volume definition in radiotherapy for lung, head and neck, genitourinary and brain tumours were performed. FDG-PET has a significant impact on GTV and PTV delineation in lung cancer. It can detect lymph node involvement and differentiate malignant tissue from atelectasis. In head and neck cancer, the value of FDG-PET is still under investigation. FDG-PET could be superior to CT and MRI in the detection of lymph node metastases and unknown primary cancer, and in the differentiation of viable tumour tissue after treatment. Therefore, FDG-PET might play an important role in GTV definition and sparing of normal tissue. The imaging of hypoxia, cell proliferation, angiogenesis, apoptosis and gene expression leads to the identification of different areas of a biologically heterogeneous tumour mass that can be individually targeted using intensity modulated radiotherapy (IMRT). A higher radiation dose may be delivered to an area with tumour hypoxia using IMRT. Grosu *et al* [27] concluded from their study that PET may be useful in three important aspects: locating the tumour and tumour margins for radiation treatment planning; identifying the biological properties of the tumour visualised by PET for optimal treatment; and evaluating the tumour response to therapy. In fact, the role of PET in monitoring therapy has been reviewed [29,30]. PET imaging allows the assessment of patients' responses to a particular therapy early in the course of treatment. However, the limitation of FDG-PET in differentiation between responders and non-responders has been brought up. Further studies to define the generally accepted cutoff values are necessary.

## COMPUTER SIMULATION OF TUMOUR RESPONSE TO RADIOTHERAPY

In the past decade, much effort has been made in the computer simulation model to help understand tumour growth and response to radiation therapy for optimising treatment planning. The In Silico Oncology Group in Greece [31-34] presented a model which is based on tumour imaging, histopathological and genetic data of patients as well as fundamental biological mechanism, i.e., tumour growth kinetics, and the linear quadratic model of cell killing by irradiation. The software was tested for validation by comparing the model prediction with clinical data before, during and after the radiotherapy course. The simulation results of glioblastoma multiforme, using different fractionated irradiation schemes, were compared in patients with different expression of insulin-like growth factor (overexpression vs. lower expression) and different status of the p53 gene (wild type vs. mutant). The simulation results were in accordance with the established clinical experience: tumours with overexpression of insulin-like growth factor were more radiosensitive. They also revealed that the trend for reduction in the number of surviving tumour cells during all schedules of radiotherapy was pronounced in the case of the tumour with wild type p53, which was more radiosensitive compared with the tumour with mutant p53. The model could qualitatively represent the clinical reality and produce biologically reasonable results. The adaptation of the simulation model to real clinical data to improve its clinical reliability is underway. The reality of the model depends on the input parameters, i.e., biological information of the patient, genetic profiles, and quality of the imaging system.

## CONCLUSIONS

Molecular biology is the key to individualised targeted cancer diagnosis and treatment. The future of radiation oncology lies in exploiting the genetics, or the microenvironment of the tumour. With genomic and proteomic studies as well as bioinformatics, the candidate molecule can be developed. The International Atomic Energy Agency (IAEA) [35] reported the ongoing studies world wide; Europe, the United Kingdom, Canada, Japan, Australia, and the United States aim to characterise the molecular profiles that predict normal tissue and tumour radioresponse. Among those, the GENEPI project (genetic pathways for the prediction of the effects of irradiation) launched by the European Society for Therapeutic Radiology and Oncology (ESTRO), is the most comprehensive one. In this study, the established tissue bank is linked to a detailed clinical outcome-database of patients receiving radiotherapy, both on normal tissue reactions and tumour responses, including accurate dosimetry and follow-up. This is a valuable resource for genetic research on radiation responses.
